# Bimetallic M/N/C catalysts prepared from π-expanded metal salen precursors toward an efficient oxygen reduction reaction†

**DOI:** 10.1039/c7ra12657c

**Published:** 2018-01-12

**Authors:** Akira Onoda, Yuta Tanaka, Koki Matsumoto, Minoru Ito, Takao Sakata, Hidehiro Yasuda, Takashi Hayashi

**Affiliations:** Department of Applied Chemistry, Graduate School of Engineering, Osaka University 2-1 Yamadaoka, Suita Osaka 565-0871 Japan onoda@chem.eng.osaka-u.ac.jp thayashi@chem.eng.osaka-u.ac.jp; Research Center for Ultra-High Voltage Electron Microscopy, Osaka University Ibaraki 567-0047 Japan

## Abstract

Nonprecious metal electrocatalysts are being explored as alternatives to platinum-group metal electrocatalysts for the oxygen reduction reaction (ORR) which is required for cathode materials in fuel cells. Herein, we describe a new method for preparing bimetallic nitrogen-containing carbon catalysts with high ORR activity using π-expanded M(salen) precursors. The M/N/C and bimetallic FeM/N/C ORR catalysts were obtained by pyrolysis of a mixture of a carbon support (Vulcan XC-72R) and the metal complex as a precursor. The bimetallic FeCu catalyst prepared from Fe and Cu complexes with the *N*,*N*′-bis(2-hydroxy-1-naphthylidene)-1,2-phenylenediamine ligand (2NAPD) is found to have an onset potential of 0.87 V, which is positively shifted by 50 mV from that of the catalyst prepared from the monometallic Fe(2NAPD) complex. The FeCu/N/C catalyst promotes efficient four-electron reduction in the ORR. High-resolution transmission electron microscopy studies reveal that both Fe and Cu metals together with pyridinic nitrogen species are highly dispersed within the carbonaceous structure in FeCu/2NAPD@VC, suggesting that the N-coordinated Fe and Cu sites promote efficient four-electron reduction of O_2_. This new methodology facilitates design of nonprecious bimetallic carbon catalysts with excellent ORR activity.

## Abstract 2

Bimetallic metal and nitrogen-containing carbon (M/N/C) catalysts were prepared by pyrolysis of a mixture of carbon support and π-expanded Fe(salen) and Cu(salen) precursors. The bimetallic catalyst promotes efficient four-electron reduction of dioxygen.

## Graphical abstract

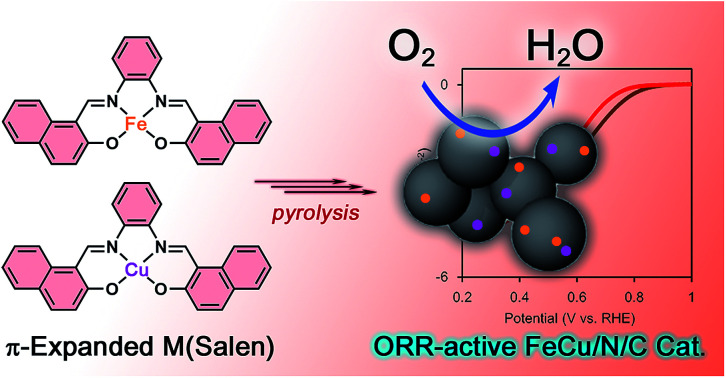

## Introduction

Electrochemical reduction of O_2_ is a pivotal reaction for improving the performance of energy conversion devices such as fuel cells, metal–air batteries, and electrolyzers.^1–3^ In particular, polymer electrolyte fuel cells (PEFCs) have been recognized as efficient energy converters enabling low emissions and low environmental impact. Precious metal group (PMG) catalysts have been the most widely used catalysts for the cathodic oxygen reduction reaction (ORR) in PEFCs.^4–6^ However, the high cost and low abundance of precious metals have been the main obstacles facing widespread commercialization of PEFCs. Thus, extensive investigations have been reported for development of alternative low-cost non-precious metal catalysts (NPMCs).^7–9^

Catalysts including a first row transition metal, such as Fe, Cu, or Co, and nitrogen atoms embedded within a carbonaceous structure are known as M/N/C catalysts and have been generally considered to represent promising alternatives to PGM catalysts, because the M/N/C catalysts have been shown to promote high levels of ORR activity while having suitable durability for use in PEFCs.^10–15^ Pyrolyzed transition metals such as Fe, Cu, and Co in combination with macrocyclic ligands adsorbed on carbon supports have been proven to improve ORR activity and stability under acidic conditions, which is a requirement for PEFCs.^16,17^ A series of low-cost nitrogen-containing precursors such as phenanthroline,^11^ polypyrrole,^10^ and polyaniline^12^ have been utilized in preparation of M/N/C catalysts. The exact chemical structures of the metal-containing active sites in the M/N/C catalysts prepared by pyrolysis remain under debate,^18,19^ although a metal and nitrogen-containing active site structure has been proposed as a catalytic site on the basis of data obtained from various spectroscopic techniques.^20–23^ It is believed that metals coordinated by the N-groups play important roles in enhancement of the ORR activity of M/N/C catalysts.^11,24^

Inspired by cytochrome c oxidase, which catalyzes O_2_ reduction using a unique bimetallic Fe and Cu active site with ultimate efficiency, a number of examples of construction of bimetallic M/N/C catalysts containing first row transition metals as active sites have been reported.^25–32^ In the enzyme, the active site containing heme iron and a copper ion is known to efficiently promote four-electron reduction of a bound O_2_ molecule to water. Thus, M/N/C catalysts prepared from two precursors with two metal ions have been investigated in attempts to improve the ORR activity.^33–35^

Previously, we reported preparation of Fe/N/C catalysts having high ORR activity by systematically tuning an aromatic framework of Fe(salen) complex precursors.^36^ We demonstrated that the designed Fe(salen) derivatives with various aromatic moieties such as 1NAPD and 2NAPD ligands have higher thermal stability, which affects the annulation process of the complexes during pyrolysis, thereby leading to higher ORR activity. In this work, we describe preparation and characterization of new bimetallic FeM/N/C catalysts *via* pyrolysis of the π-expanded metal salen complexes M(2NAPD) and measurement of their ORR activity. It is found that combining the M(2NAPD) precursors, in particular Fe and Cu, leads to a positive shift of onset potentials of the ORR activity of the pyrolyzed bimetallic M/N/C catalysts.

## Results and discussion, experimental

### Preparation of FeM/N/C catalysts

**Fig. 1:**
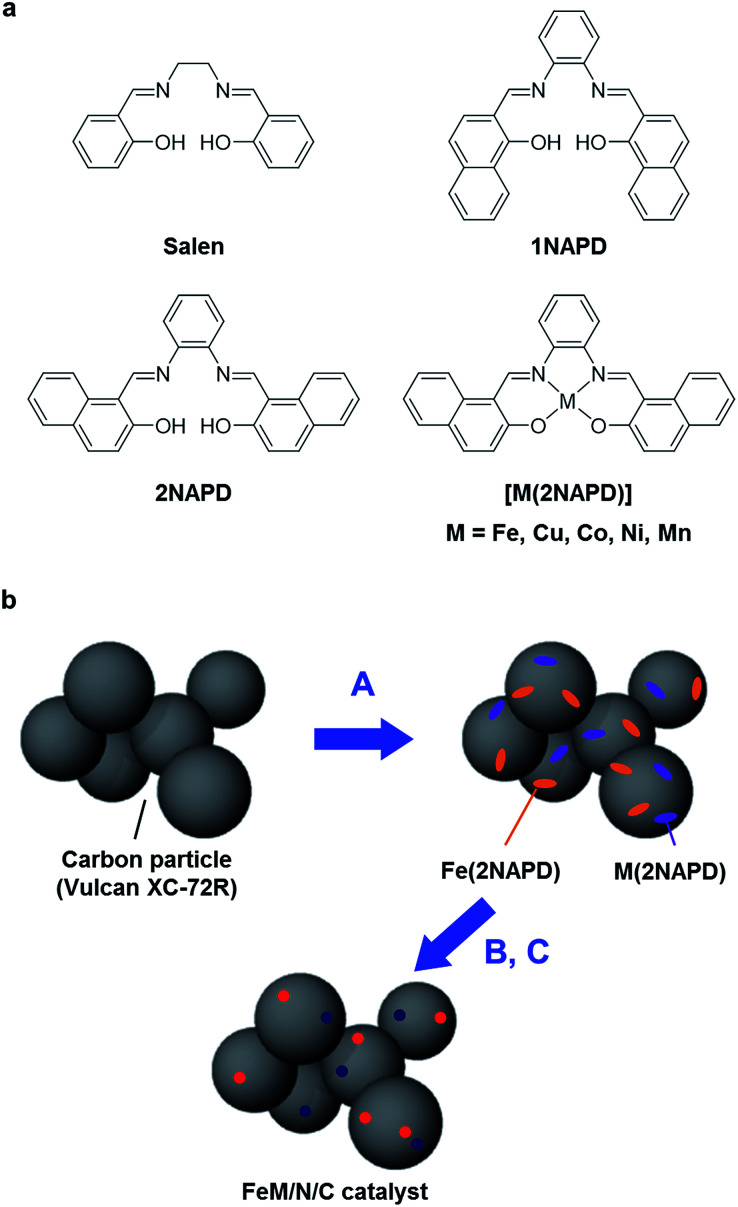
(a) Structures of salen, 1NAPD, 2NAPD ligand and M(2NAPD) complexes. (b) The scheme for preparation of bimetallic FeM/N/C catalysts. (A) Mixing of Fe(2NAPD) complex (orange) and M(2NAPD) complex (purple) with carbon materials, (B) pyrolysis at 1000 °C under N_2_ atmosphere, and (C) treatment with H_2_SO_4aq_ to remove metal impurities and to prepare the FeM/N/C catalysts containing the iron (red) and other metal (dark blue) active sites.

A series of M(2NAPD) complex precursors with expanded aromatic ligand frameworks were synthesized according to the literature (Fig. 1a).^37–40^ The thermal decomposition temperature (*T*_D_) value of *N*,*N*′-bis(2-hydroxy-1-naphthylidene)-1,2-phenylenediaminoiron(iii) chloride (Fe(2NAPD)) containing the expanded aromatic ring framework is 417 °C. This is shifted more than 84 °C above the *T*_D_ value of Fe(salen). Therefore, we prepared additional corresponding metal complexes Cu(2NAPD), Co(2NAPD), Ni(2NAPD) and Mn(2NAPD) as catalyst precursors. The thermogravimetric analysis of the precursors indicates that the aromatic rings in the ligand frameworks also enhance the thermal stability of Cu(2NAPD), Co(2NAPD), Ni(2NAPD), and Mn(2NAPD) complexes during pyrolysis (Table 1, Fig. S1†).

**Table 1:** Characterization and electrochemical activity of M/N/C catalysts

M/N/C catalyst^a^	*T*_D_ a (°C)	BET (m^2^ g^−1^)	Elemental analysis		XPS (N 1s)				*E*_onset_ c (V)	*n* d
			M^b^ (wt%)	N (wt%)	Pyridinic (%)	Pyrrolic (%)	Graphitic (%)	N-oxide (%)		
Fe/2NAPD@VC	417	143	0.9	0.8	24.9	13.4	50.2	11.5	0.82	3.6
Cu/2NAPD@VC	396	422	1.1	0.4	21.2	2.8	69.5	6.6	0.76	3.3
Co/2NAPD@VC	435	294	1.2	0.7	16.0	6.5	70.5	7.0	0.74	3.2
Ni/2NAPD@VC	445	362	0.8	0.9	10.1	9.0	71.8	9.2	0.55	2.7
Mn/2NAPD@VC	264	328	0.2	0.6	17.8	6.8	63.1	12.3	0.54	2.7
2NAPD@VC	230	801	—	0.3	8.9	5.6	79.3	6.2	0.53	2.6

a Decomposition temperature of M(2NAPD) precursors demined by TG-DTA.

b Determined by ICP-AES.

c The potential at *I* = 0.05 mA cm^−2^ in RDE.

d The number of electrons transferred during O_2_ reduction calculated by Koutecky–Levich plots at 0.3 V.

The M/N/C electrocatalysts were prepared by pyrolysis of the mixture of M(2NAPD) complexes and carbon black Vulcan XC-72R (VC, Cabot, USA) (Fig. 1b). One or two types of the M(2NAPD) complex dissolved in CHCl_3_ were vigorously mixed with VC and the solvent was removed to afford the precursor. The obtained precursor was preheated to 300 °C for 1 h and then incubated at 300 °C for 2 h under constant N_2_ gas flow. The carbon materials were immediately pyrolyzed at 1000 °C for 2 h under constant N_2_ gas flow for carbonization. The pyrolyzed powder was ground and leached in an acidic solution (0.5 M H_2_SO_4_) to remove metal species such as metal oxides and unincorporated metal species. After washing twice with an excess of water and drying, Fe/2NAPD@VC, Cu/2NAPD@VC, Co/2NAPD@VC, Ni/2NAPD@VC, Mn/2NAPD@VC, FeCu/2NAPD@VC, FeCo/2NAPD@VC, FeNi/2NAPD@VC, and FeMn/2NAPD@VC were produced. These materials were characterized by elemental analysis, inductively coupled plasma atomic emission spectrometry (ICP-AES), X-ray diffraction (XRD), and Raman spectroscopy, and X-ray photoelectron spectroscopy (XPS).

### ORR activity

**Fig. 2:**
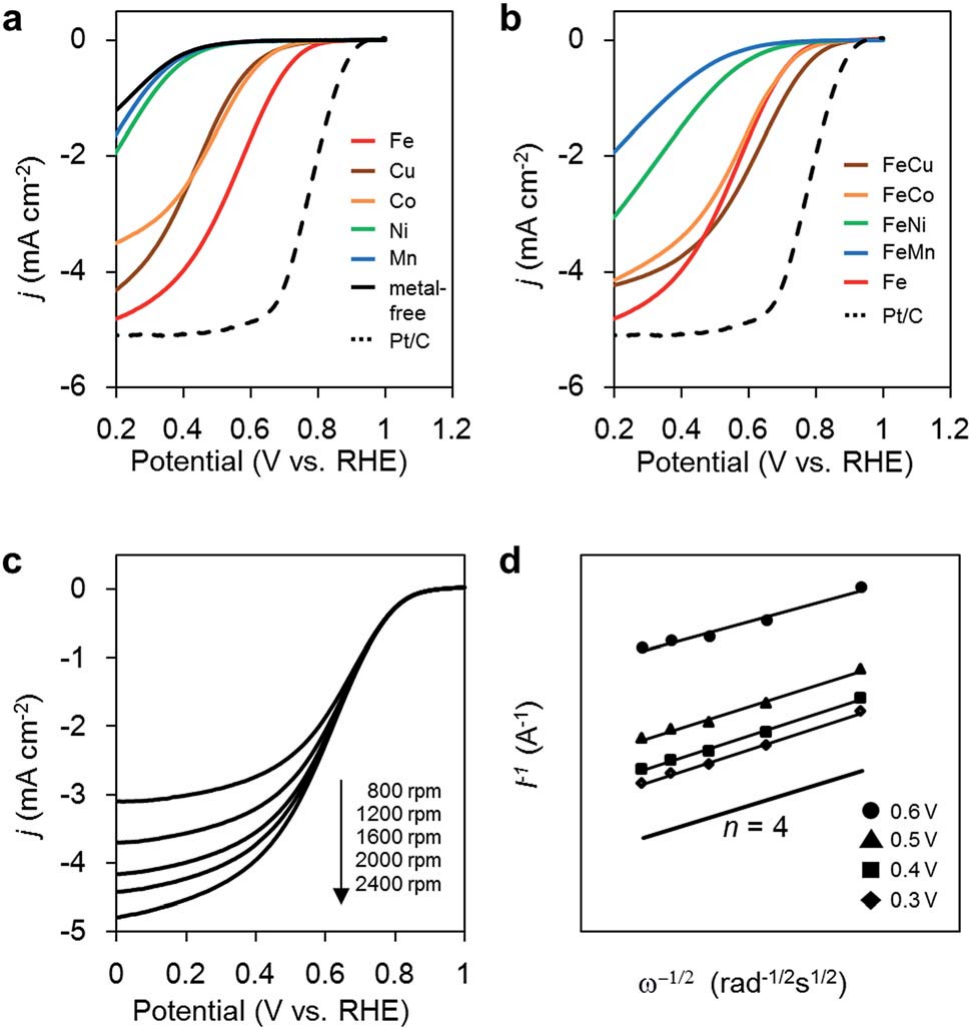
(a) ORR polarization curves of M/N/C catalysts; Fe/2NAPD@VC (red), Cu/2NAPD@VC (brown), Co/2NAPD@VC (orange), Ni/2NAPD@VC (green), Mn/2NAPD@VC (blue), metal-free 2NAPD@VC (black), and Pt/C catalyst, TEC10V30E (dashed black). (b) ORR polarization curves of FeM/N/C catalysts; FeCu/2NAPD@VC (brown), FeCo/2NAPD@VC (orange), FeNi/2NAPD@VC (green), FeMn/2NAPD@VC (blue), Fe/2NAPD@VC (red), and Pt/C catalyst, TEC10V30E (dashed black). In O_2_-saturated 0.1 M HClO_4_ solution at 5 mV s^−1^ with 2000 rpm. (c) ORR polarization curves of FeCu/2NAPD@VC with various rotation rates. (d) Koutecky–Levich plots at different potentials for FeCu/2NAPD@VC.

The ORR activities of the M/N/C and bimetallic FeM/N/C catalysts were determined using a rotating disk electrode at different rotation rates in a medium of O_2_-saturated 0.1 M HClO_4_ at pH 1 (Fig. 2). M/N/C and FeM/N/C catalysts prepared from the M(2NAPD) precursors show significant cathodic current during O_2_ reduction, indicating high levels of ORR activity. The percentage of the number of electrons transferred during O_2_ reduction (*n*) was determined from Koutecky–Levich plots for each carbon catalyst (Tables 1, 2, Fig. 2c, d, S2 and S3†).

**Table 2:** Characterization and electrochemical activity of FeM/N/C catalysts

FeM/N/C catalyst	BET (m^2^ g^−1^)	Elemental analysis			XPS (N 1s)				*E*_onset_ b (V)	*n* c
		Fe^a^ (wt%)	M^a^ (wt%)	N (wt%)	Pyridinic (%)	Pyrrolic (%)	Graphitic (%)	N-oxide (%)		
FeCu/2NAPD@VC	447	1.2	0.7	0.8	20.0	9.7	62.3	8.0	0.87	3.9
FeCo/2NAPD@VC	631	0.4	0.2	0.5	18.9	6.1	61.7	13.3	0.83	3.6
FeNi/2NAPD@VC	896	1.1	0.5	0.7	14.0	16.2	54.3	15.6	0.76	3.5
FeMn/2NAPD@VC	1095	0.6	0.1	0.5	17.5	17.6	52.0	12.8	0.69	3.5

a Determined by ICP-AES.

b The potential at *I* = 0.05 mA cm^−2^ in RDE.

c The number of electrons transferred during O_2_ reduction calculated by Koutecky–Levich plots at 0.3 V.

First, in the case of the monometallic M/N/C catalyst, as we reported previously, the onset potentials of the Fe/N/C catalysts shift positively for the catalyst prepared from the Fe(salen) complex with the π-expanded ligand framework. In particular, Fe/2NAPD@VC has a significantly shifted onset potential, suggesting that introduction of aromatic rings into the ligand framework of the precursor positively shifts the onset potential in Fe/2NAPD@VC relative to Fe/salen@VC.^36^ Among the M/N/C catalysts prepared in this work, Fe/2NAPD@VC has the most shifted positive onset potentials (0.82 V) relative to the onset potentials of other catalysts including Cu/2NAPD@VC, Co/2NAPD@VC, Ni/2NAPD@VC, and Mn/2NAPD@VC (Table 1). The average number of electrons transferred during O_2_ reduction is 3.6 for Fe/2NAPD@VC, generally indicating occurrence of four electron reduction. Both Cu/2NAPD@VC and Co/2NAPD@VC also show moderate activity for four electron reduction. In contrast, Ni/2NAPD@VC and Mn/2NAPD@VC promote two electron reduction as well as metal-free 2NAPD@VC.

On the basis of the electrochemical result showing high electrocatalytic ORR activity for Fe/2NAPD@VC, we prepared bimetallic FeM/N/C catalysts using Fe(2NAPD) and other M(2NAPD) complexes as precursors and their electrocatalytic properties were analyzed. The onset potentials of FeCu/2NAPD@VC, FeCo/2NAPD@VC, FeNi/2NAPD@VC, and FeMn/2NAPD@VC are 0.87 V, 0.83 V, 0.76 V, and 0.69 V, respectively. Interestingly, the onset potential (0.87 V) of FeCu/2NAPD@VC exhibits remarkable positive shifts relative to the onset potential of Fe/2NAPD@VC (0.82 V). In addition, FeCo/2NAPD@VC also has a slightly shifted onset potential (0.83 V). Furthermore, the *n* value of FeCu/2NAPD@VC (3.9) is higher than that of Fe/2NAPD@VC (3.6). The results clearly indicate that the bimetallic catalyst FeCu/2NAPD@VC promotes four electron reduction of O_2_ and has higher activity relative to that of monometallic Fe/2NAPD@VC and Cu/2NAPD@VC. We also found that the 1 : 1 ratio of the Fe and Cu 2NAPD complexes has the best activity (Fig. S4a†). Furthermore, the bimetallic Fe/Cu carbon catalyst prepared from the mixture of simple Fe and Cu salen complexes has a negatively shifted onset potential of 0.77 V (Fig. S4b†). These findings indicate that the ORR catalysts prepared from the mixed M(2NAPD) precursors have a synergistic effect provided by bimetallic active sites, which efficiently proceed four-electron reduction. Since the onset potential for ORR shifts negatively to 0.84 V after 100 cycles, the durability of FeCu/2NAPD@VC was not excellent (Fig. S9†).

### Characterization of the M/N/C and FeM/N/C catalysts

**Fig. 3:**
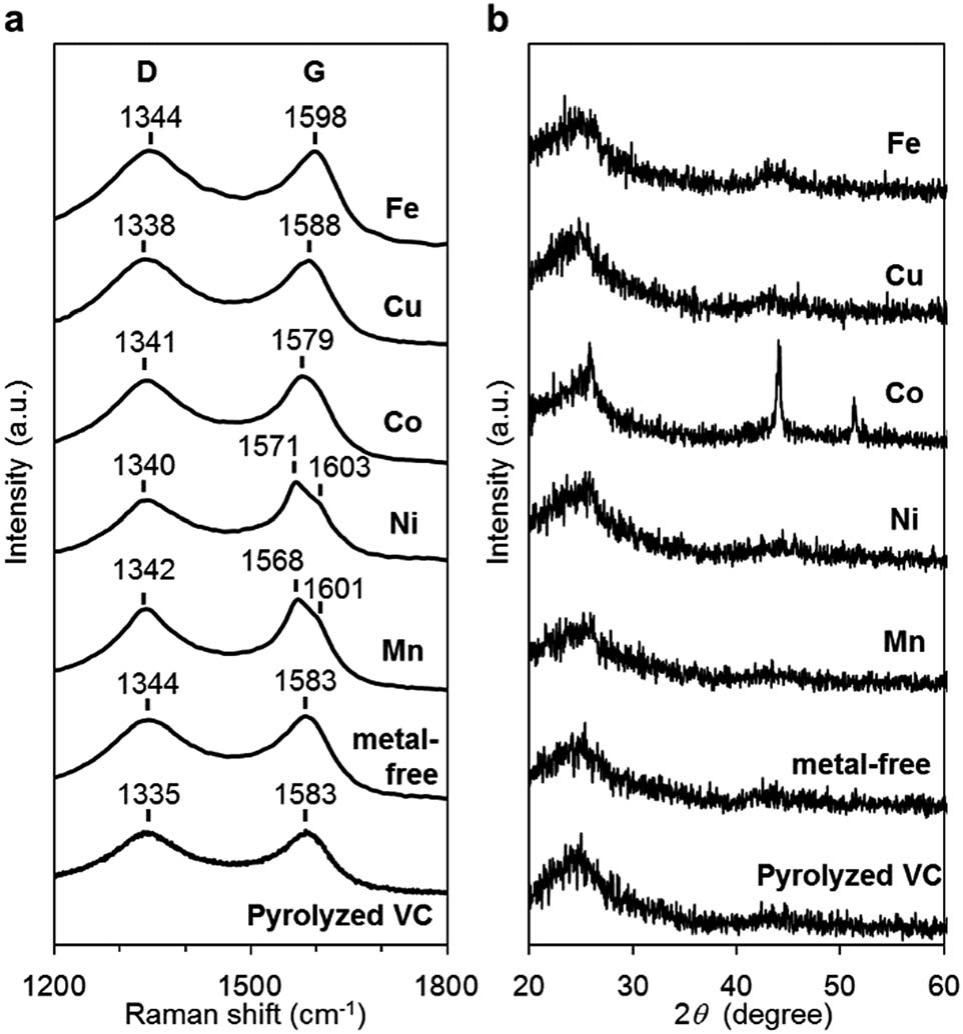
Characterization of the M/N/C catalysts prepared from M(2NAPD) precursors. (a) Raman spectra and (b) XRD patterns of Fe/2NAPD@VC, Co/2NAPD@VC, Ni/2NAPD@VC, Mn/2NAPD@VC, 2NAPD@VC, and pyrolyzed VC.

**Fig. 4:**
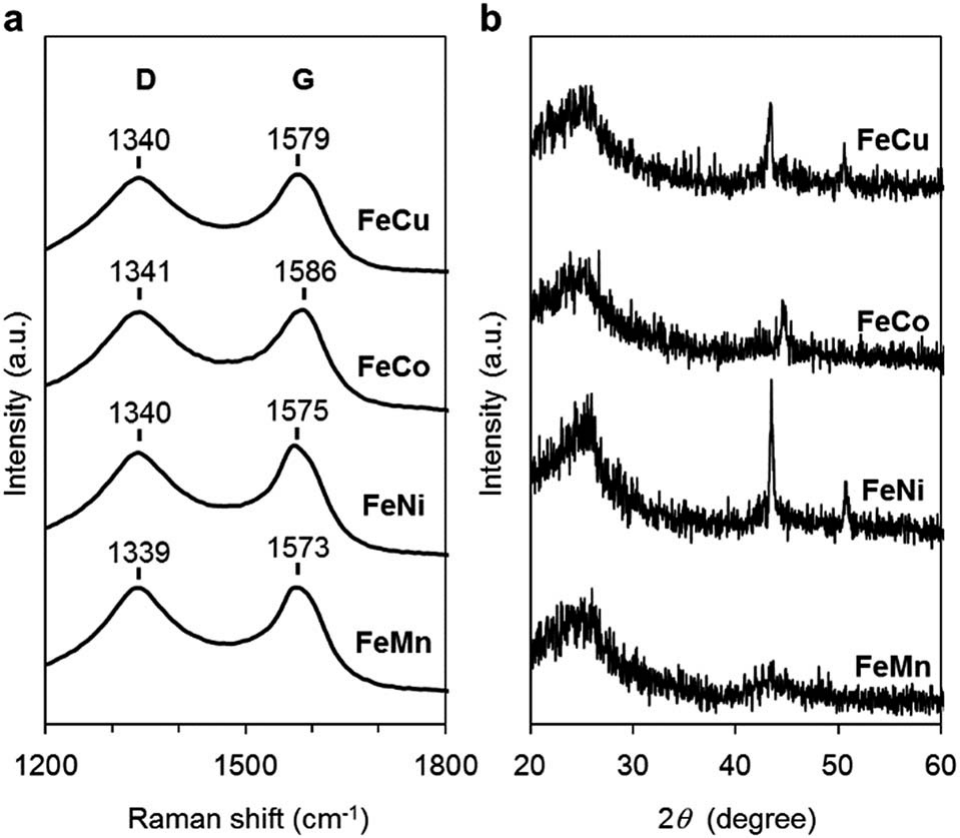
Characterization of the FeM/N/C catalysts prepared from M(2NAPD) precursors. (a) Raman spectra and (b) XRD patterns of FeCu/2NAPD@VC, FeCo/2NAPD@VC, FeNi/2NAPD@VC, and FeMn/2NAPD@VC.

The carbon materials in the monometallic M/N/C and bimetallic FeM/N/C catalysts were first analyzed by Raman spectroscopy (Fig. 3a and 4a). It is known that a disordered carbon structure has significantly different spectra with a D (disorder) band in the vicinity of 1355 cm^−1^. Graphitized carbons give rise to a G (graphite) band in the vicinity of 1580 cm^−1^, which is assignable to in-plane displacement of carbon strongly coupled in the hexagonal sheets.^41,42^ The Raman spectra of the M/N/C and bimetallic FeM/N/C catalysts presenting typical D- and G-bands indicate that the disordered carbonaceous structure is included in these catalysts. The integrated intensity ratio *I*_D_/*I*_G_ for the D-band and G-band is widely used to quantify defects in graphitic materials. The *I*_D_/*I*_G_ ratio for all monometallic M/N/C and bimetallic FeM/N/C catalysts is approximately 1.1, suggesting that all catalysts include similar graphitic and disordered structural content in the carbonaceous materials. The nitrogen-doped graphene has been known to have D′ peak near 1600 cm^−1^.^43–45^ The G band peaks for the M/N/C catalysts are located at 1600 cm^−1^, which represents a shift from the peak at 1583 cm^−1^ for VC without nitrogen atom and for VC pyrolyzed with the 2NAPD ligand. This result indicates that nitrogen atoms are doped in the carbon framework in accordance with the metal species.

X-ray diffraction (XRD) experiments were performed to confirm each carbonaceous structure of the M/N/C and FeM/N/C catalysts prepared from the M(2NAPD) complexes and the chemical composition of the metal species (Fig. 3b and 4b). In general, powder samples of carbons in an amorphous structure provide diffraction peaks for (002) (2*θ* = *ca.* 26.0°), and (101) (2*θ* = *ca.* 44.0°).^46,47^ The carbon catalysts were found to exhibit a strong and broad peak at *ca.* 26.0° and a weak and broad peak at 44.0°. In addition, the peaks for Co/2NAPD@VC (2*θ* = 43.9° and 51.2°), FeCu/2NAPD@VC (2*θ* = 43.3° and 50.5°), FeCo/2NAPD@VC (2*θ* = 45.1°), and FeNi/2NAPD@VC (2*θ* = 43.6° and 50.8°) indicate that Co metal,^48^ FeCu,^49^ FeCo,^50^ and FeNi alloys^51^ are generated in each catalyst.

The content of metal and N atoms in the M/N/C and the FeM/N/C catalysts was determined by elemental analysis with ICP-AES measurements. The elemental compositions of the Fe/N/C and FeM/N/C catalysts are summarized in Tables 1 and 2 together with other properties. The content of the metal ranges between 1.2–0.1%. Therefore, the metals of the precursors are retained in the carbonaceous structure after treatment of an aqueous H_2_SO_4_ solution at 80 °C to remove the metal nanoparticles and metal oxides. In addition, the nitrogen content of the M/N/C catalysts is higher than that of the 2NAPD@VC catalyst prepared from 2NAPD without metal (0.3%). This suggests that the larger amount of the nitrogen species is incorporated into the carbonaceous structure by the pyrolysis of the metal salen precursors.

**Fig. 5:**
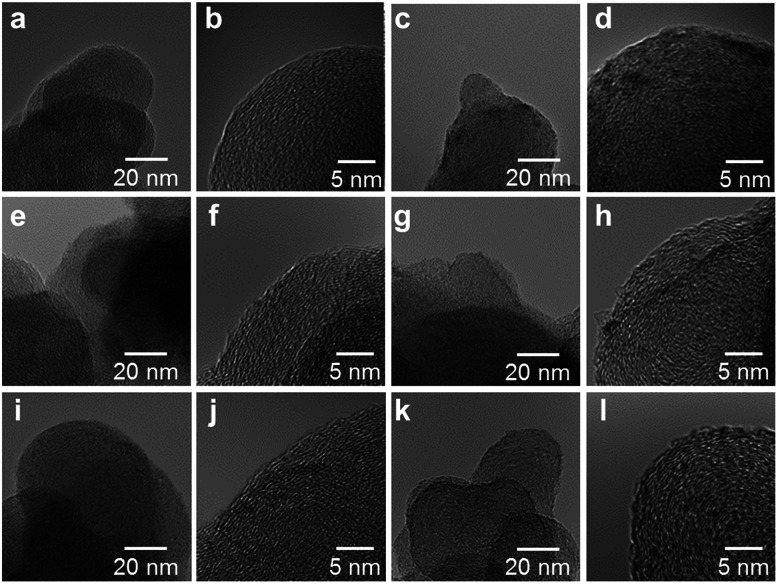
HRTEM images of (a, b) 2NAPD@VC, (c, d) Fe/2NAPD@VC, (e, f) Cu/2NAPD@VC, (g, h) Co/2NAPD@VC, (i, j) Ni/2NAPD@VC, and (k, l) Mn/2NAPD@VC (magnification = 300k and 1000k). The metal content of each of the samples determined by EDS analysis with an electron probe of 25 nm in the area with 300k magnification is (c) Fe: 0.79 wt%, (e) Cu: 0.80 wt%, (g) Co: 0.19 wt%, (i) Ni: 0.72 wt%, and (k) Mn: 0.54 wt%.

**Fig. 6:**
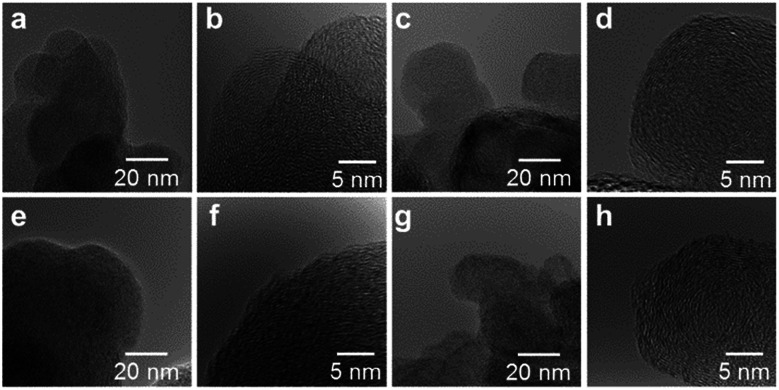
HRTEM images of (a, b) FeCu/2NAPD@VC, (c, d) FeCo/2NAPD@VC, (e, f) FeNi/2NAPD@VC, and (g, h) FeMn/2NAPD@VC (magnification = 300k and 1000k). The metal content of each of the samples determined by EDS analysis with an electron probe of 25 nm in the area with 300k magnification is (a) Fe: 0.31 wt%, Cu: 0.18 wt%, (c) Fe: 0.33 wt%, Co: 0.16 wt%, (e) Fe: 0.33 wt%, Ni: 0.16 wt%, and (g) Fe: 0.40 wt%, Mn: 0.27 wt%.

High resolution transmission electron microscope (HRTEM) images of the M/N/C and FeM/N/C catalysts were obtained to determine the nanostructures of the catalyst (Fig. 5 and 6). The M/N/C catalysts are found to have a spherical form with diameters ranging from 40 to 70 nm, which are retained in the structure of VC. We confirmed the existence of each metal in the carbon structure by performing chemical composition analyses using EDS (Energy dispersive X-ray spectrometry). The data are in good accordance with the data obtained from the ICP-AES measurements. Therefore, the main components of metals are highly dispersed in the carbon structure in each catalyst. In the case of Co/2NAPD@VC, FeCo/2NAPD@VC, and FeNi/2NAPD@VC, we also observed nanoparticles of Co metal, FeCo, and FeNi alloys, respectively in HRTEM images (Fig. S6†). In contrast, such nanoparticle of FeCu alloys were not observed at all in the FeCu/N/C catalyst (Fig. S7†), which shows a similar trend observed in Fe/2NAPD@VC and Cu/2NAPD@VC. Considering the existence of the FeCu alloy structure in XRD analysis, the results indicate that highly dispersed FeCu alloys are generated in FeCu/2NAPD@VC, which has the highest activity in four electron reduction of O_2_.

### XPS measurements

**Fig. 7:**
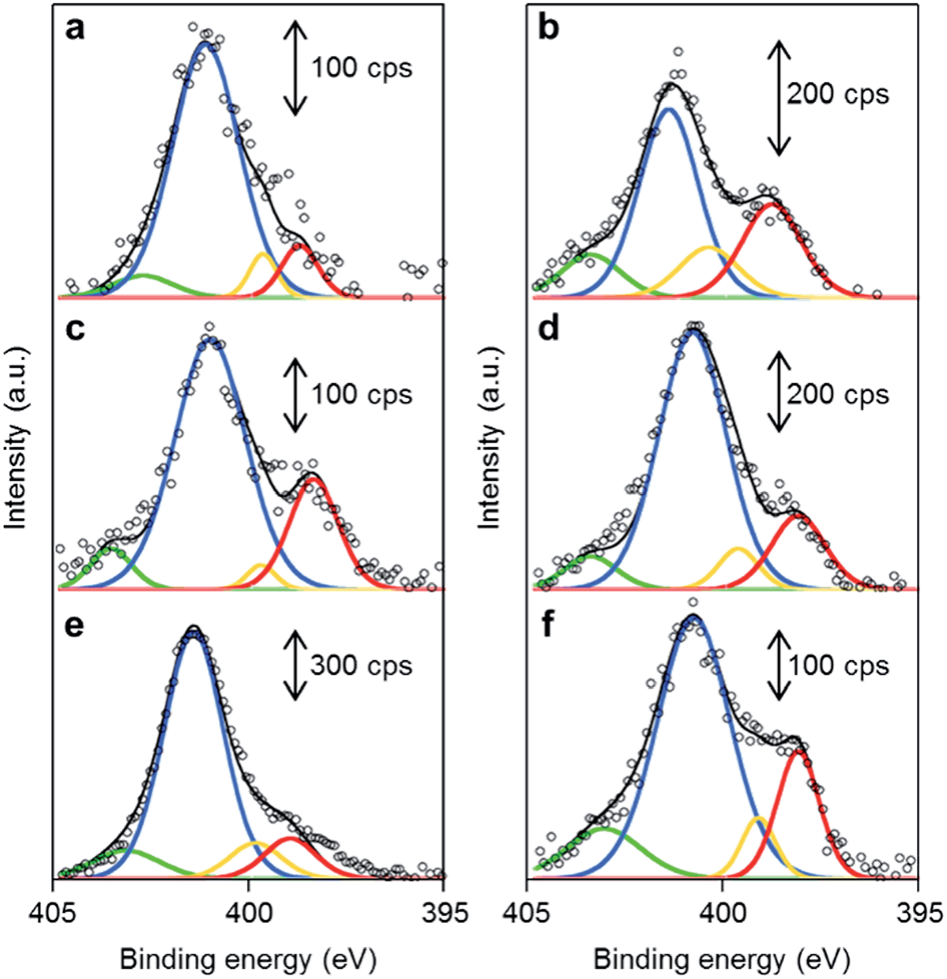
The distribution of pyridinic N, pyrrolic N, graphitic N and N-oxide in M/N/C catalysts. XPS spectra of (a) 2NAPD@VC, (b) Fe/2NAPD@VC, (c) Cu/2NAPD@VC, (d) Co/NAPD@VC, (e) Ni/2NAPD@VC, and (f) Mn/2NAPD@VC in the N 1s region with fitted peaks of pyridinic-N (red), pyrrolic-N (yellow), graphitic-N (blue), and N-oxide (green).

**Fig. 8:**
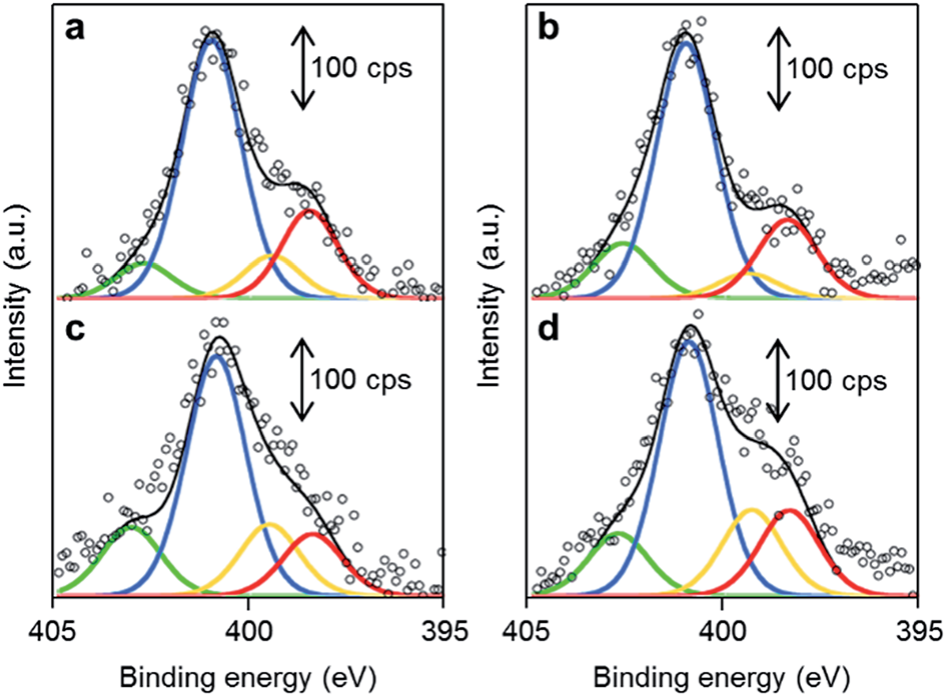
The distribution of pyridinic N, pyrrolic N, graphitic N and N-oxide in FeM/N/C catalysts. XPS spectra of (a) FeCu/2NAPD@VC, (b) FeCo/2NAPD@VC, (c) FeNi/2NAPD@VC, and (d) FeMn/2NAPD@VC in the N 1s region with fitted peaks of pyridinic-N (red), pyrrolic-N (yellow), graphitic-N (blue), and N-oxide (green).

The chemical structures of the nitrogen atoms in the M/N/C and FeM/N/C catalysts were determined by X-ray photoelectron spectroscopy (XPS) measurements (Fig. 7 and 8). Four types of nitrogen species are confirmed by the N 1s peaks in the range between 398.0 and 404.0 eV.^52,53^ The N 1s spectra were deconvoluted into four different nitrogen species including pyridinic N (398.0–398.9 eV), pyrrolic N (399.5–400.4 eV), graphitic N (400.5–402.0), and N-oxide groups (N^+^–O^−^) at binding energies higher than 402.0 eV. The content is summarized in Tables 1 and 2. Interestingly, the content of pyridinic N of Fe/2NAPD@VC (24.9%) and Cu/2NAPD@VC (21.2%) is higher than that of the catalyst prepared from 2NAPD (8.9%). This indicates that the pyridinic moiety in the carbonaceous structure is important to bind the metal active sites in the catalysts. This is consistent with the reported evidence that metals coordinated by the N-groups play important roles in enhancement of the ORR activity of M/N/C catalysts.^11,24^ We found that the nitrogen content (0.8 wt%) and the pyridinic nitrogen content (20%) for FeCu/2NAPD@VC are higher than those of other catalysts. Since the catalysts are treated in acid during the preparation, only the carbon-encapsulated metal or metal alloy nanoparticles remain in the carbonaceous structure. Therefore, we expect that the N-coordinated Fe and Cu sites, which are highly dispersed in the carbonaceous structure, are the active sites promoting efficient four-electron reduction of O_2_ in the FeCu/2NAPD@VC catalyst.

## Conclusions

This work demonstrates a new method for improving the ORR activity of bimetallic M/N/C catalysts using mixed metal precursors containing π-expanded ligand frameworks. The bimetallic FeM/N/C catalysts were prepared by pyrolysis of the mixture of carbon support and the M(2NAPD) precursors. The catalyst prepared using the Fe(2NAPD) complex with π-expanded ligand framework exhibits a remarkable positive shift in the onset potential for ORR relative to catalysts prepared from the M(2NAPD) complex (M = Cu, Co, Ni, Mn). By mixing these heterometallic precursors, we found that ORR activity can be significantly improved. In particular, FeCu/2NAPD@VC has a positively-shifted onset potential for ORR with excellent four-electron O_2_ reduction activity. Both Fe and Cu metals together with pyridinic nitrogen species are highly dispersed within the carbonaceous structure in FeCu/2NAPD@VC, suggesting that the N-coordinated Fe and Cu sites promote efficient four-electron reduction of O_2_. These findings prove that our approach of using aromatic ligand frameworks for metal precursor complexes contributes to enhancement of ORR activity of non-precious bimetallic M/N/C catalysts which are appropriate for incorporation into fuel cells and metal–air battery applications.

## Experimental section

### Catalyst preparation

Carbon black Vulcan XC-72R (VC, Cabot, USA) was used as a carbon support. Pt/C (29 wt%) catalyst (TEC10V30E) purchased from Tanaka Kikinzoku Kogyo K.K. was used as received. Electrocatalysts were prepared from M(2NAPD) complexes and VC by pyrolysis in N_2_ flow. The electrocatalysts, which are abbreviated as M/2NAPD@VC and FeM/2NAPD@VC, were prepared as follows: Fe(2NAPD) (42 mg, 84 μmol) was dissolved in a minimum volume of CHCl_3_ and mixed with a powder of VC (30 mg). The suspension was vigorously vortexed and sonicated for 30 min. After removal of the solvent, the residue was used as a precursor for an electrocatalyst. Fe(2NAPD) (42 μmol) and M(2NAPD) (42 μmol) (M = Cu, Co, Ni, Mn) were used for FeM/2NAPD@VC. The precursor was placed on an alumina boat (length: 80 mm, width: 16 mm, height: 10 mm), and then inserted into a quartz tube (diameter 50 mm, length 800 mm). The quartz tube was installed in a hinge split tube furnace (Koyo Thermo Systems Co. Ltd., KTF045N1). The precursor was preheated from ambient temperature to 300 °C for 1 h under N_2_ flow (0.2 L min^−1^), and incubated for 2 h. The precursor was then heated to 1000 °C for 1 h immediately, and incubated for 2 h. The temperature of the sample inside the furnace was recorded with a thermocouple equipped with a data logger (CHINO Corporation, MC3000). After cooling, the pyrolyzed catalyst was ground and further treated in a 0.5 M H_2_SO_4_ solution at 80 °C for 3 h to leach out impurities such as metal oxides and then washed with excess volumes of deionized water twice. The dried carbon catalyst was used for the experiments. Other carbon catalysts (Cu/2NAPD@VC, Co/2NAPD@VC, Ni/2NAPD@VC, Mn/2NAPD@VC, 2NAPD@VC, FeCu/2NAPD@VC, FeCo/2NAPD@VC, FeNi/2NAPD@VC, and FeMn/2NAPD@VC) were obtained using the same protocol with the iron complex.

### Physicochemical characterization

The decomposition temperatures of M(2NAPD) complexes were determined by thermogravimetry-differential thermal analysis (TG-DTA) using a Mac Science TG-DTA TMA DSC with a heating rate of 10 °C min^−1^ under an N_2_ stream in a platinum pan. Metal content of each of the M/N/C catalysts and the FeM/N/C catalysts was determined by inductively coupled plasma atomic emission spectroscopy (ICP-AES) using a SHIMAZDU ICPS-7510 system. Specific surface areas were obtained using a Quantachrome NOVA 4200e Surface Analyzer and calculated by the Brunauer–Emmett–Teller (BET) method. The Raman spectra were obtained using a JASCO NRS-3100 instrument with a 532 nm laser. X-ray diffraction (XRD) patterns of the samples were obtained using an X-ray diffractometer (Rigaku, SmartLab) equipped with a Cu Kα source. High-resolution transmission electron microscopy (HRTEM) observations and chemical composition analyses were carried out using a HITACHI HF-2000 field emission TEM operated with an accelerating voltage of 200 kV. The chemical composition of each sample was analyzed by EDS (NORAN Instruments). The analyses were carried out using an electron probe approximately 25 nm in diameter. The characteristic X-ray of metals was collected with an ultra-thin window X-ray detector at a high take-off angle of 68 degrees. The morphology of the catalysts were observed using a JEOL JSM-6335 field emission scanning electron microscope (FE-SEM) operated at an accelerating voltage of 20 kV. XPS measurements were performed on a KRATOS AXIS-165x (SHIMADZU) system, equipped with a Mg Kα X-ray source. Individual chemical components of the N 1s binding energy region were fitted to the spectra after a Tougaard-type background subtraction.

### Electrochemical measurement

A rotating ring-disk electrode with a glassy carbon disk electrode (*ϕ* = 5 mm) and platinum ring was used for evaluation of the carbon catalysts. Electrode rotation rates were controlled using a Pine Instruments AFMSRCE rotator with a Pine MSRX motor controller. The electrode was polished to mirror flat with alumina powder (50 nm) before use. The catalyst ink included a mixture of 12.0 mg of catalyst ((Fe/2NAPD@VC, Cu/2NAPD@VC, Co/2NAPD@VC, Ni/2NAPD@VC, Mn/2NAPD@VC, 2NAPD@VC, FeCu/2NAPD@VC, FeCo/2NAPD@VC, FeNi/2NAPD@VC, or FeMn/2NAPD@VC)), and 50 μL of 5 wt% Nafion® solution (Sigma-Aldrich), and 950 μL of isopropanol. The ink was vortexed and sonicated in an ultrasonic bath at 100 W at 35 kHz for 30 min. Then 10 μL of the catalyst ink was loaded onto the surface of the electrode and dried.

Electrochemical tests were carried out on a potentiostat (ALS, electrochemical analyzer model 610B) using a typical three-electrode system, with platinum wire as an counter electrode and Ag/AgCl as a reference electrode. The potential difference between Ag/AgCl and RHE was calculated and the value is 0.258 V in a 0.1 M HClO_4_ solution. The scan rate for all measurements was 5 mV s^−1^ from −0.258 to 0.742 V *versus* the Ag/AgCl reference electrode. Before each potential scan, the electrolyte of the 0.1 M HClO_4_ solution was saturated with O_2_ for at least 30 min, and O_2_ purging was continued during the electrochemical experiments. The measured current was subtracted from the background current at the N_2_-saturated electrolyte. The number of electrons transferred during O_2_ reduction was calculated using the Koutecky–Levich equation (eqn (1) and (2))^54^

1 *I*^−1^ = *I*_K_^−1^ + *I*_L_^−1^

2 *I*_L_ = 0.620*nFAD*_0_^2/3^*ω*^1/2^*ν*^−1/6^*C*_0_

where *I*, *I*_K_, and *I*_L_ represent the measured, kinetically-controlled, and diffusion-limited currents, respectively. *n* is the number of exchanged electrons, *ω* is the angular frequency of rotation, *ω* = 2π*f*/60, *f* is the RDE rotation rate in rpm, *F* is the Faraday constant (96 485C mol^−1^), *D*_0_ is the diffusion coefficient of O_2_ in 0.1 M HClO_4_ solution (1.9 × 10^−5^ cm^2^ s^−1^), *ν* is the kinematic viscosity of electrolyte (9.87 × 10^−3^ cm^2^ s^−1^), and *C*_0_ is the concentration of O_2_ (11.8 × 10^−6^ mol cm^−3^).

## Conflicts of interest

There are no conflicts to declare.

## Supplementary Material

RA-008-C7RA12657C-s001
