# Supplementary material for: Bimetallic M/N/C catalysts prepared from π-expanded metal salen precursors toward an efficient oxygen reduction reaction
Source: RSC Adv. 2018 Jan 12;8(6):2892–9. doi: 10.1039/c7ra12657c (PMC9077469; doi:10.1039/c7ra12657c)
Supplement: RA-008-C7RA12657C-s001 [file RA-008-C7RA12657C-s001.pdf]

Electronic Supplementary Material (ESI) for RSC Advances.
This journal is © The Royal Society of Chemistry 2018

Supporting Information

Bimetallic M/N/C catalysts prepared from π-expanded metal salen precursors toward an efficient oxygen reduction reaction

Akira Onoda,*[a] Yuta Tanaka,[a] Koki Matsumoto,[a] Minoru Ito,[a] Takao Sakata,[b] Hidehiro Yasuda,[b] and Takashi Hayashi*[a]

[a]*Department of Applied Chemistry, Graduate School of Engineering,Osaka University, 2-1 Yamadaoka, Suita, Osaka 565-0871, Japan.*
[b]*Research Center for Ultra-High Voltage Electron Microscopy, Osaka University, Ibaraki, 567-0047, Japan.*

EXPERIMENTAL SECTION

Materials. All reagents were used without purification. All solvents were dried with molecular sieves 3A before use. The 2NAPD ligand was synthesized according to our previous report.[S1]

Synthesis of M(2NAPD) complexes. Metal ion was inserted to the 2NAPD ligand by mixing 1 equivalent of metal salt ($FeCl_3$, $Cu(OAc)_2$, $Co(OAc)_2$, $Ni(OAc)_2$ or $Mn(OAc)_2$) in 300 mL of ethanol/acetone (1/1, v/v). The reaction mixture was refluxed for 2 h, and cooled to room temperature. The precipitates were collected by filtration, washed with a small portion of ethanol, and dried to yield the products.

[Fe(2NAPD)Cl]. Yield: 69%; UV-vis (DMSO, nm): 334, 397, 453; ESI-TOF MS (positive mode) *m/z* calcd for $C_{28}H_{18}FeN_2O_2$ $[M]^+$ 470.07, found 470.07.

[Cu(2NAPD)]. Yield: 87%; UV-vis (DMSO, nm): 336, 429, 466; ESI-TOF MS (positive mode) *m/z* calcd for $C_{28}H_{18}CuN_2O_2$ $[M+Na]^+$ 500.06, found 500.05.

[Co(2NAPD)]. Yield: 92%; UV-vis (DMSO, nm): 346, 420; ESI-TOF MS (positive mode) *m/z* calcd for $C_{28}H_{18}CoN_2O_2$ $[M]^+$ 473.07, found 473.06.

[Ni(2NAPD)]. Yield: 96%; UV-vis (DMSO, nm): 331, 387, 484; ^{1}H NMR (400 MHz, DMSO-d_6) δ 9.42 (2H, s, N=C*H*), 8.56 (2H, d, *J* = 8.6 Hz, 8-*H*), 8.49–8.46 (2H, m, *J* = 6.5 Hz, 4′-*H*), 7.86 (2H, d, 4-*H*), 7.81 (2H, d, *J* = 7.7 Hz, 5-*H*), 7.58 (2H, t, *J* = 7.2 Hz, 8.6 Hz, 7-*H*), 7.38–7.34 (4H, m, *J* = 6.5 Hz, 7.2 Hz, 7.7 Hz, 3′-*H*, 6-*H*), 7.15 (2H, d, *J* = 9.3 Hz, 3-*H*).

[Mn(2NAPD)]. Yield: 83%; UV-vis (DMSO, nm): 375, 453, 482; ESI-TOF MS (positive mode) *m/z* calcd for $C_{28}H_{18}FeN_2O_2$ $[M]^+$ 469.07, found 469.07.

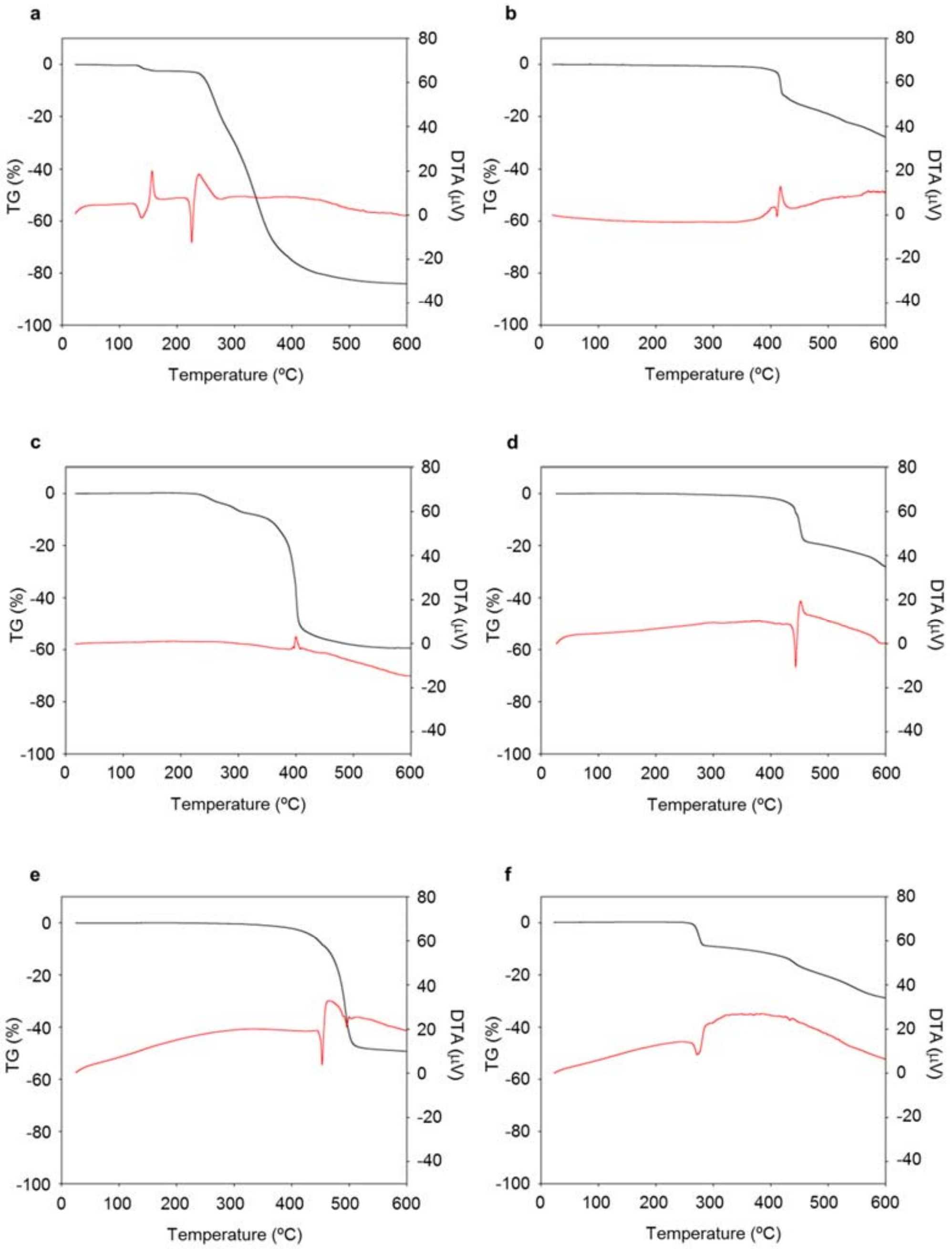

Fig. S1 Thermogravimetric-differential thermal analysis of M(2NAPD) precursors. Thermal gravimetric analysis curves (black) and differential thermal analysis curves (red) of M(2NAPD) precursors. (a) 2NAPD, (b) Fe(2NAPD)Cl, (c) Cu(2NAPD), (d) Co(2NAPD), (e) Ni(2NAPD), and (f) Mn(2NAPD).

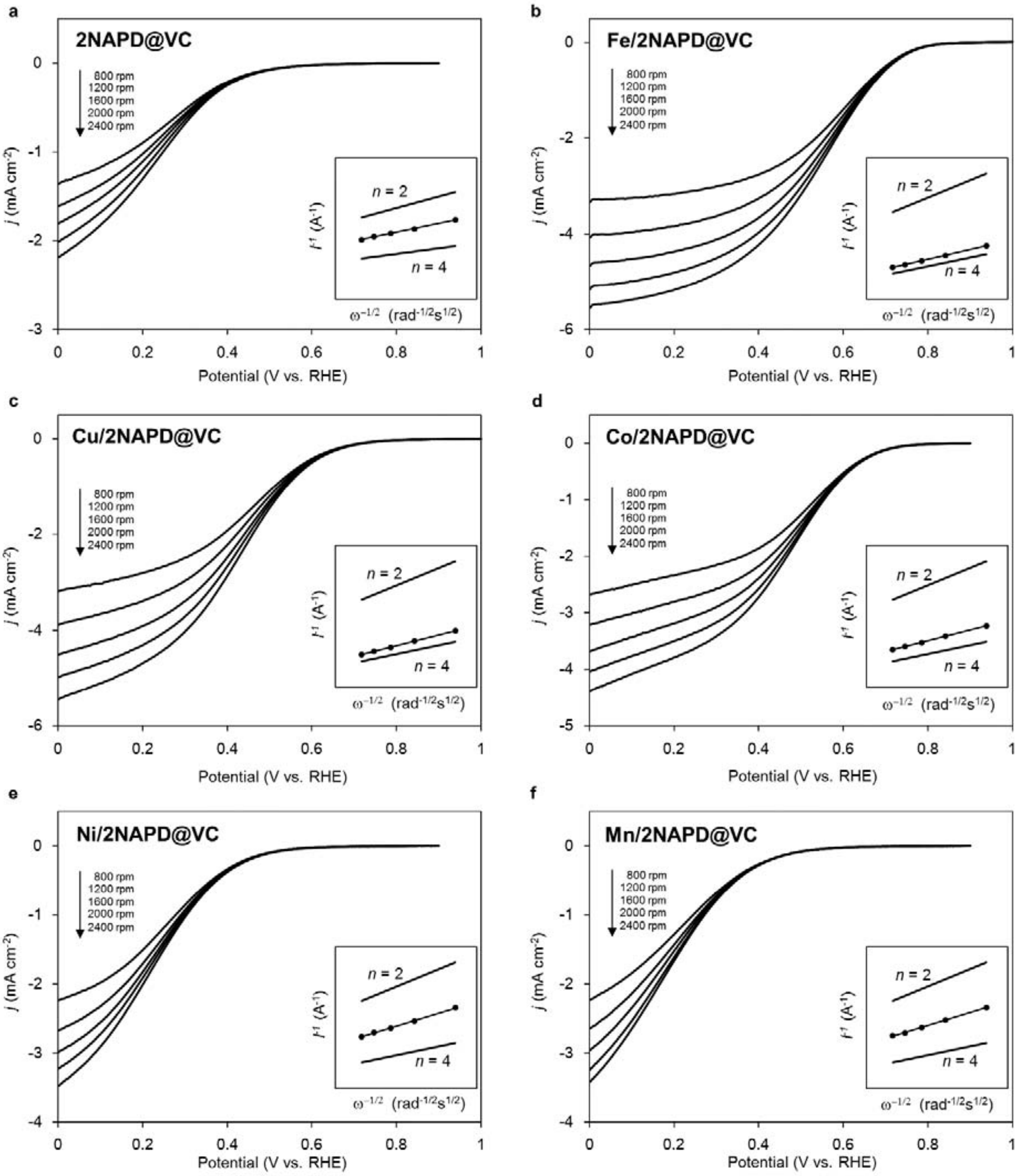

Fig. S2 ORR polarization curves of (a) 2NAPD@VC, (b) Fe/2NAPD@VC, (c) Cu/2NAPD@VC, (d) Co/2NAPD@VC, (e) Ni/2NAPD@VC, and (f) Mn/2NAPD@VC collected at a sweep rate of 5 mV/s in O_2-saturated 0.1 M $HClO_4$. The rotation rates are 800, 1200, 1600, 2000, and 2400 rpm. The insets represent the Koutecky-Levich plots at 0.3 V vs RHE.

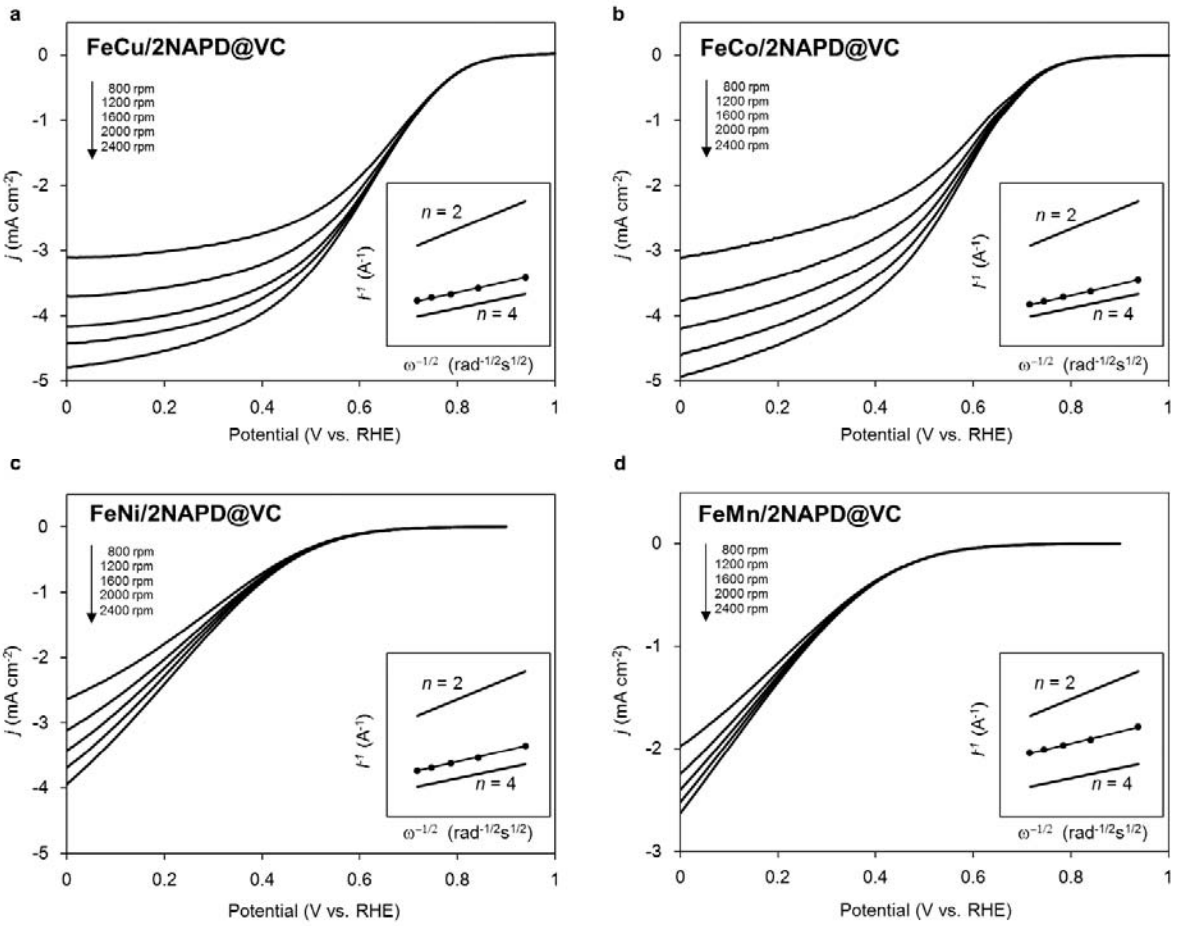

Fig. S3 ORR polarization curves of (a) FeCu/2NAPD@VC, (b) FeCo/2NAPD@VC, (c) FeNi/2NAPD@VC, and (d) FeMn/2NAPD@VC collected at a sweep rate of 5 mV/s in O_2-saturated 0.1 M $HClO_4$. The rotation rates are 800, 1200, 1600, 2000, and 2400 rpm. The insets represent the Koutecky-Levich plots at 0.3 V vs RHE.

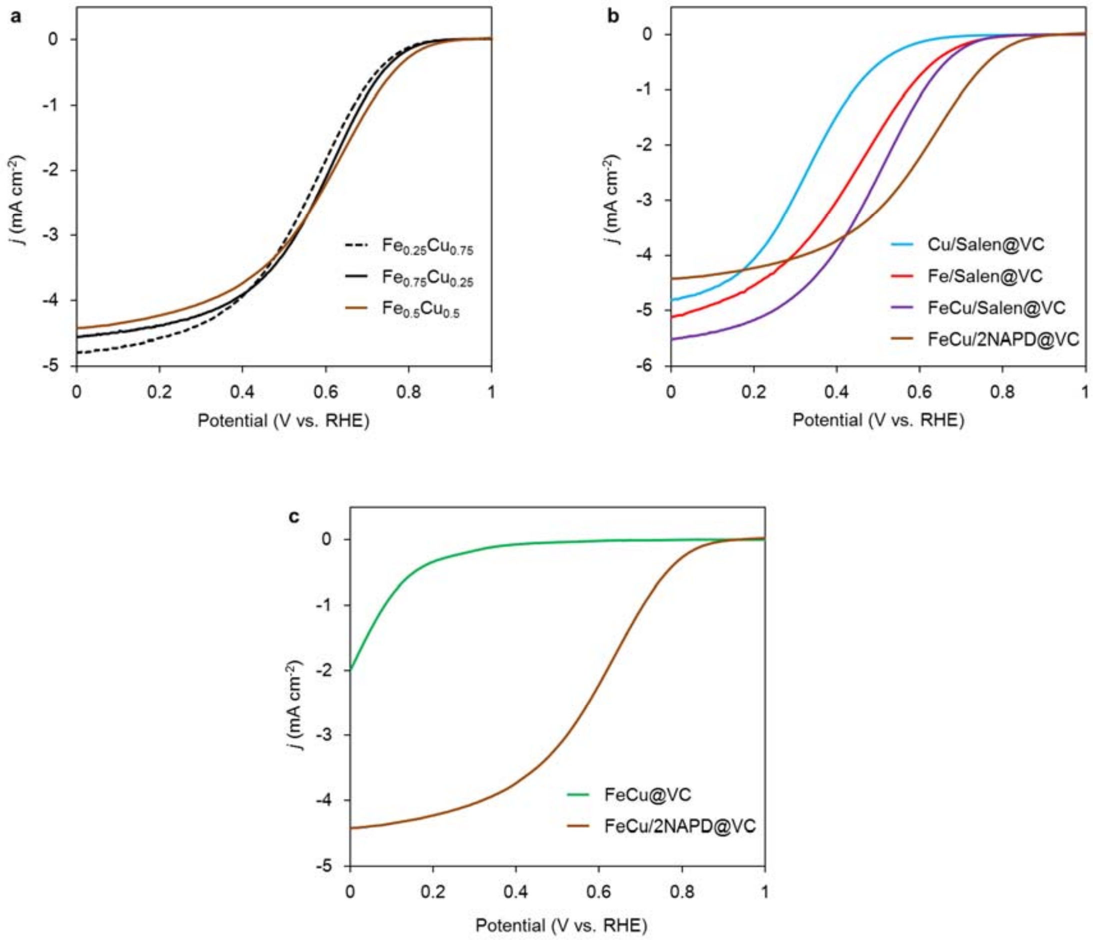

Fig. S4 ORR polarization curves of FeCu/2NAPD@VC catalyst and the reference samples. (a) Influence of the ratio of Fe precursor to Cu precursor. $Fe_{0.25}Cu_{0.75}$/2NAPD@VC (dashed black line), $Fe_{0.75}Cu_{0.25}$/2NAPD@VC (black line), and FeCu/2NAPD@VC (brown). $Fe_{0.25}Cu_{0.75}$/2NAPD@VC and $Fe_{0.75}Cu_{0.25}$/2NAPD@VC catalysts were prepared from 21 μmol of Fe precursor and 63 μmol of Cu precursor, 63 μmol of Fe precursor and 21 μmol of Cu precursor. The onset potentials are 0.83 V ($Fe_{0.25}Cu_{0.75}$/2NAPD@VC), 0.84 V ($Fe_{0.75}Cu_{0.25}$/2NAPD@VC), and 0.87 V (FeCu/2NAPD@VC). (b) Influence of the π-expanded salen ligand (2NAPD). Cu/Salen@VC (blue), Fe/Salen@VC (red), FeCu/Salen@VC (purple), the catalysts prepared from M(Salen) complexes with simple salen ligand, and FeCu/2NAPD@VC (brown). The onset potentials are 0.66 V (Cu/Salen@VC), 0.73 V (Fe/Salen@VC), 0.77 V (FeCu/Salen@VC), and 0.87 V (FeCu/2NAPD@VC). (c) Influence of 2NAPD ligand. FeCu@VC (green), and FeCu/2NAPD@VC (brown). The FeCu@VC catalyst was prepared from 42 μmol of $FeCl_3$ and 42 μmol of Cu acetate. The onset potentials are 0.45 V (FeCu@VC), and 0.87 V (FeCu/2NAPD@VC).

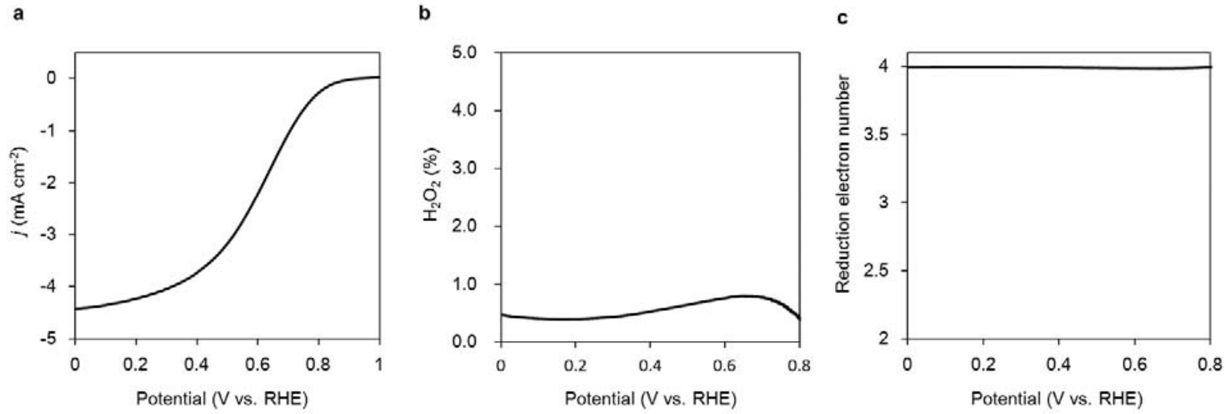

Fig. S5 RDE and RRDE. (a) ORR polarization curve, (b) the percentage of generated H_2O_2, and the number of electrons transferred during O_2 reduction in O_2-saturated 0.1 M $HClO_4$ solution at 5 mV/s with 2000 rpm of FeCu/2NAPD@VC. The RRDE collection efficiency (N) was calibrated in 0.1 M KNO_3 with 10 mM $K_3Fe(CN)_6$ electrolyte. The measured N value is 0.26 in our system. The H_2O_2 yield was calculated by following the equation: $H_2O_2(\%) = 2\ I_r / (N \times |I_d| + I_r) \times 100$, where I_r represents the ring current and I_d represents the disk current. The ring potential was set to 1.1 V vs RHE in RRDE measurement.

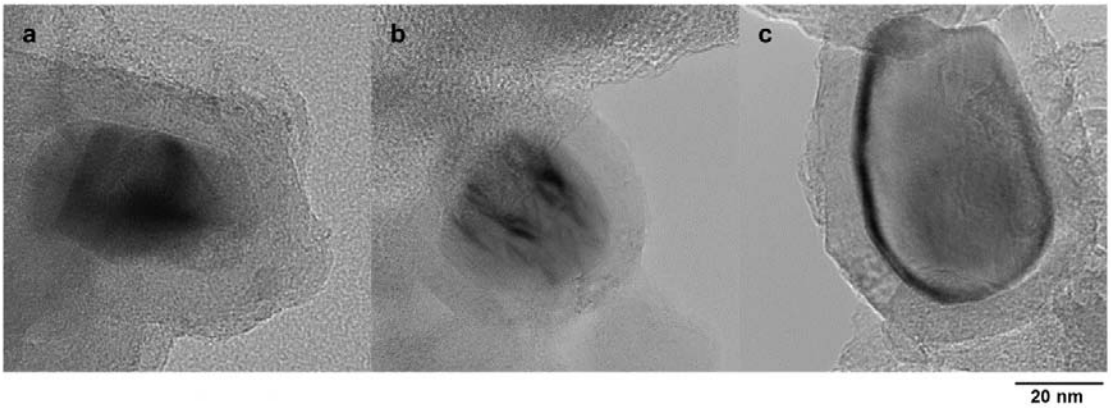

Fig. S6 HRTEM images of metal particles in (a) Co/2NAPD@VC (b) FeCo/2NAPD@VC, and (c) FeNi/2NAPD@VC (magnification = 300k). EDS analysis with an electron probe of 25 nm in the area with 300k magnification revealed that the ratio of iron to metal ((b) Co or (c) Ni) in metal particles was 1:1, respectively.

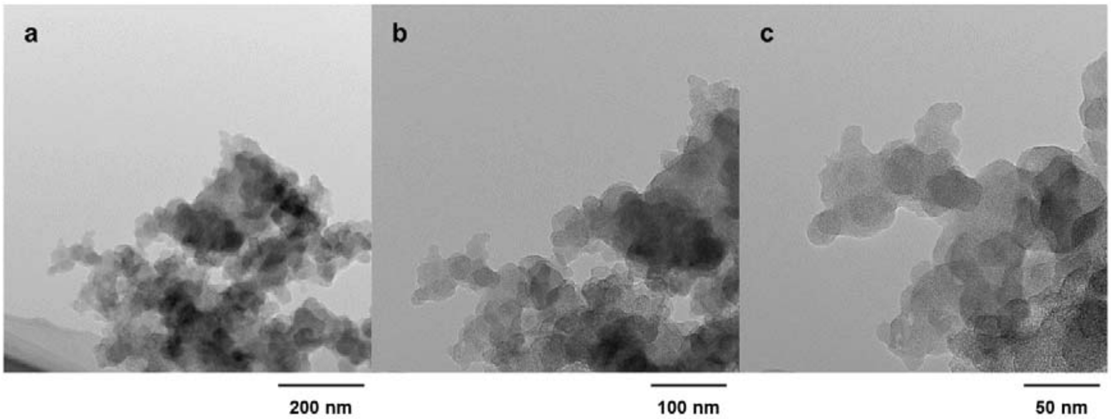

Fig. S7 HRTEM images of FeCu/2NAPD@VC with low magnification. (a) 30k, (b) 50k, and (c) 100k.

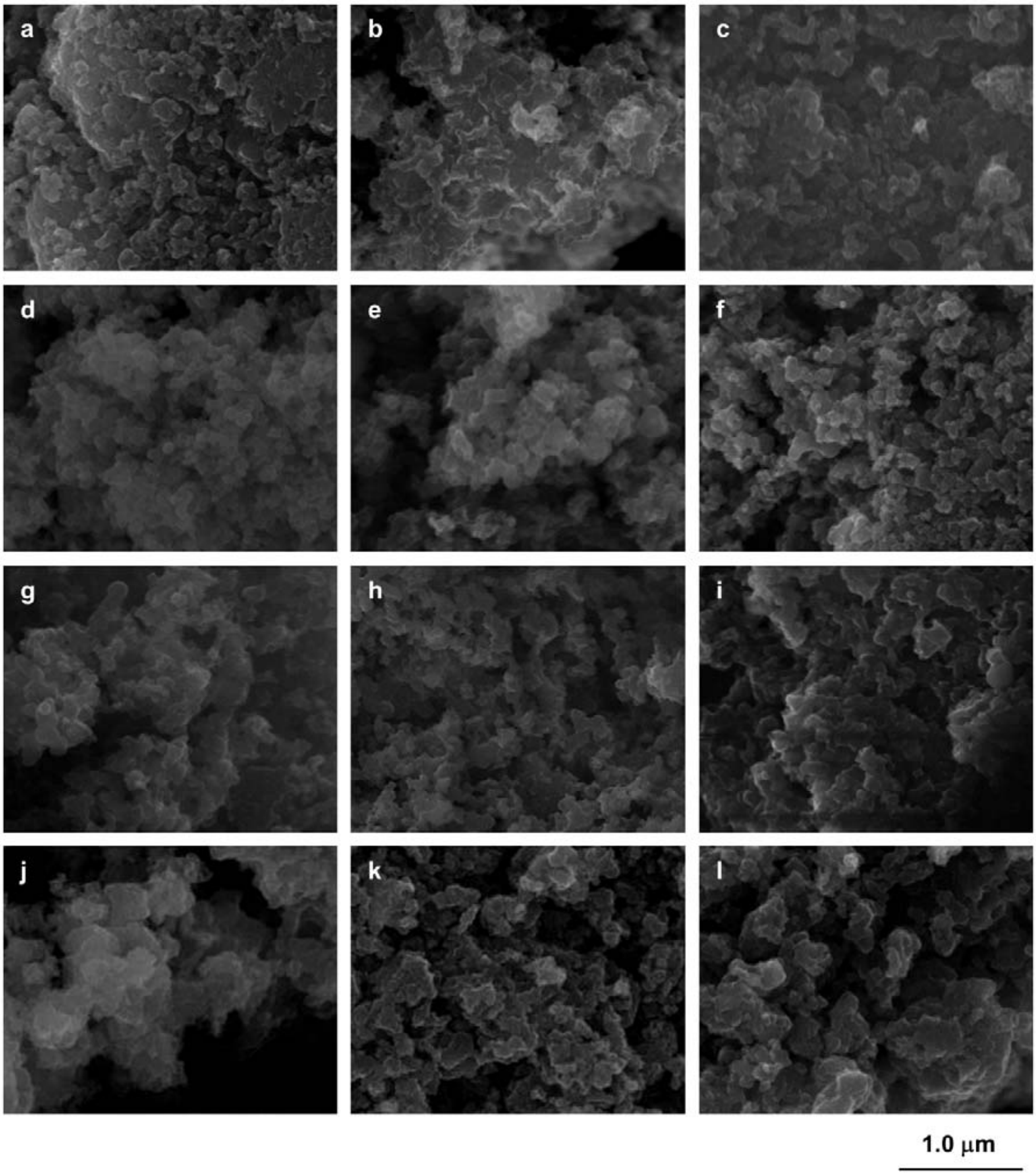

Fig. S8 SEM images of the M/N/C catalysts and the FeM/N/C catalysts. (a) 2NAPD@VC, (b) Fe/2NAPD@VC, (c) Cu/2NAPD@VC, (d) Co/2NAPD@VC, (e) Ni/2NAPD@VC, (f) Mn/2NAPD@VC, (g) FeCu/2NAPD@VC, (h) FeCo/2NAPD@VC, (i) FeNi/2NAPD@VC, (j) FeMn/2NAPD@VC, (k) $Fe_{0.25}Cu_{0.75}$/2NAPD@VC, and (l) $Fe_{0.75}Cu_{0.25}$/2NAPD@VC.

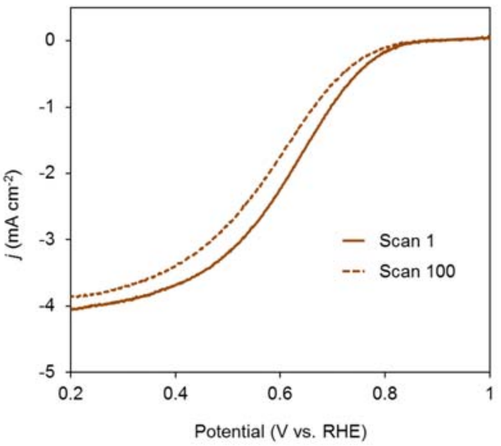

Fig. S9 ORR polarization curves of FeCu/2NAPD@VC before and after 100 times scans in O_2-saturated 0.1 M $HClO_4$ solution at 5 mV·s^{-1} with 2000 rpm.

Reference

1. Y. Tanaka, A. Onoda, S. Okuoka, T. Kitano, K. Matsumoto, T. Sakata, H. Yasuda and T. Hayashi, *ChemCatChem*, *in press*.
